# Impact of Lifestyle Interventions on the Progression of Diabetic Retinopathy in Patients Diagnosed With Diabetes Mellitus: A Systematic Review

**DOI:** 10.7759/cureus.82411

**Published:** 2025-04-17

**Authors:** Ali Zain Abden M AlShammari, Faisal F Alhamad, Zeinab E Elbashir Abdelgadir, Hanaa M AlShammari, Mubark Alahmadi, Khalid F Alharbi

**Affiliations:** 1 College of Medicine, Al-Rayan National College of Medicine, Madinah, SAU; 2 Ophthalmology, Al-Rayan National College of Medicine, Madinah, SAU; 3 Ophthalmology, Ohud Hospital, Madinah, SAU

**Keywords:** diabetic retinopathy, lifestyle intervention, non-proliferative diabetic retinopathy (npdr), ophthalmology, prevention ophthalmology, proliferative diabetic retinopathy (pdr), risk factors for diabetic retinopathy

## Abstract

Diabetes mellitus is a major global health issue, with rising prevalence and complications such as diabetic retinopathy (DR). Effective prevention strategies, including glycemic, lipid, and blood pressure control, are essential. Lifestyle interventions - particularly diet and physical activity - are increasingly recognized for their role in mitigating DR progression.

The review followed Preferred Reporting Items for Systematic Reviews and Meta-Analyses (PRISMA) guidelines and searched PUBMED, Web of Science, Cochrane Library, and ScienceDirect for studies published between 2010 and 2024. Included studies assessed the impact of dietary changes and physical activity on DR severity, retinal vascular complications, glycemic control (HbA1c), visual acuity, and inflammatory biomarkers. Statistical analysis, including risk ratios and heterogeneity, was performed using RevMan version 5.4.1 (The Cochrane Collaboration, Oxford, UK), and the risk of bias in randomized controlled trials (RCTs) was assessed accordingly.

Across 10 studies involving various lifestyle interventions, combined diet and exercise significantly improved glycemic control (lower HbA1c), lipid profiles, inflammation markers, and slowed DR progression. Interventions such as culturally tailored education, e-coaching, and long-term physical activity were particularly effective. Sleep and gut microbiota also emerged as influential lifestyle factors. However, a few studies showed no significant long-term impact, highlighting the dominant role of cumulative HbA1c. HbA1c levels were reduced by up to 1.1% (e.g., from 7.2% to 6.1%, p < 0.05), and the progression rate of DR was reduced by 47% in long-term follow-up studies (HR = 0.53, 95% CI: 0.29-0.99).

Lifestyle interventions - especially those combining a healthy diet and physical activity - effectively reduce DR progression in patients with diabetes by improving metabolic control and reducing inflammation. These findings support the integration of personalized, technology-assisted lifestyle programs into diabetes care for DR prevention. However, long-term glycemic control remains a critical factor.

## Introduction and background

Diabetes mellitus, commonly referred to as diabetes, is a chronic metabolic disorder characterized by insulin resistance, where the body is unable to effectively use the insulin it produces, leading to high blood sugar levels, also known as hyperglycemia [1,2]. Diabetes mellitus primarily includes two main types - Type 1 and Type 2 - though other forms, such as gestational diabetes mellitus (GDM) and secondary diabetes caused by other medical conditions, also exist. In Type 1 diabetes, the body’s immune system mistakenly attacks and destroys the insulin-producing cells in the pancreas. In Type 2 diabetes, the body either becomes insulin resistant or the pancreas fails to secrete sufficient insulin [3]. The inability to either secrete or properly use insulin results in high and uncontrolled blood sugar levels, which can lead to serious health complications over time [4]. One such complication is diabetic retinopathy (DR), a progressive microvascular condition characterized by retinal vascular damage [4].

DR is one of the most serious diabetes-related complications, causing hemorrhages, exudates, neovascularization, and, in advanced stages, vision loss [5]. Any diabetic person is at high risk of DR, with the risk increasing with the duration of diabetes and poor blood sugar control [6]. Over the years, many types of interventions have been developed, many of which are pharmacological, including anti-vascular endothelial growth factor (anti-VEGF) therapy, corticosteroids, and laser photocoagulation [7,8]. However, in recent years, research has increasingly explored the potential of lifestyle interventions in preventing and slowing DR progression [8]. Although the impact of lifestyle modifications - particularly dietary changes and increased physical activity - on diabetes management has long been recognized, the ability of these interventions to influence DR progression remains a subject of ongoing research. Overwhelming evidence has shown that strict adherence to a healthy diet and regular physical exercise can stop or slow disease progression [6,9]. Generally, an appropriate diet is associated with improved retinal health, while regular physical exercise has been linked to improved insulin sensitivity, reduced systemic inflammation, and lower levels of circulating proangiogenic factors [9]. Consequently, the risk of damage to the blood vessels in the retina is minimized.

This systematic review seeks to synthesize existing literature on the impact of lifestyle interventions on DR progression among individuals with diabetes. The review examines results from various types of studies, including controlled trials, cohort studies, and observational research, to assess the effectiveness of diet and exercise in managing DR. The findings of this review will be crucial, as they highlight the lifestyle interventions that have been shown to stop or minimize DR progression, thereby informing clinical practices and public health strategies.

## Review

Materials and methods

This study adopted the guidelines set forth by the Preferred Reporting Items for Systematic Reviews and Meta-Analyses (PRISMA) [10], through which five medical databases were searched for relevant scholarly publications published between 2010 and 2024. These databases included PUBMED, Web of Science, the Cochrane Library, and ScienceDirect.

A combination of keywords was used to search for relevant articles for review. General terms included: ("diabetic retinopathy" OR "diabetic eye disease") AND ("lifestyle intervention" OR "diet" OR "nutrition" OR "physical activity") AND ("progression" OR "severity" OR "incidence") AND ("randomized controlled trial" OR "cohort study" OR "longitudinal study").

Eligibility, Data Extraction, and Management

All researchers involved in this study rigorously assessed the retrieved articles for eligibility by comparing them to the pre-defined inclusion and exclusion criteria they had collectively agreed upon.

Inclusion and Exclusion Criteria

This systematic review included studies that investigated the impact of lifestyle interventions (diet and exercise) on DR progression in patients diagnosed with diabetes mellitus. Eligible studies encompassed randomized controlled trials (RCTs), cohort studies, observational studies, retrospective analyses, and longitudinal studies that assessed the effects of dietary modifications, physical activity, or combined lifestyle interventions on DR progression. The primary outcomes of interest included changes in DR severity using established grading scales, such as the ETDRS (Early Treatment Diabetic Retinopathy Study) scale, retinal vascular complications, glycemic control (HbA1c), visual acuity, and inflammatory biomarkers. Studies had to compare lifestyle interventions with standard care or other treatment approaches. Only articles published in English between 2010 and 2025 were included in this review.

Two independent reviewers (Authors A and B) screened all titles and abstracts for eligibility based on predefined inclusion and exclusion criteria. Full texts of potentially relevant studies were retrieved and assessed independently. Any discrepancies or disagreements between the two reviewers were resolved through discussion with a third reviewer (Author C) until consensus was reached.

The eligibility criteria were designed to ensure the inclusion of high-quality, relevant, and recent studies that provide meaningful insights into the impact of lifestyle interventions (diet and exercise) on DR progression. The review strictly adhered to PRISMA guidelines to maintain methodological rigor. Any disagreements among researchers were promptly resolved through inclusive discussions to reach a consensus. All relevant data were systematically retrieved and documented to ensure comprehensive coverage of the topic.

Statistical Data Analysis

To assess the methodological quality of the included studies, we used two validated tools based on study design. For cross-sectional, retrospective, prospective, and observational studies, the Newcastle-Ottawa Scale (NOS) was applied. This scale evaluates studies across three domains: selection of participants, comparability of groups, and ascertainment of exposure or outcome, assigning a maximum of nine stars to indicate quality. Studies scoring between seven and nine stars were considered high quality. For RCTs, we used the Cochrane Collaboration Risk of Bias Tool (RevMan version 5.4.1; The Cochrane Collaboration, Oxford, UK), which assesses bias across seven domains: random sequence generation, allocation concealment, blinding of participants and personnel, blinding of outcome assessment, incomplete outcome data, selective reporting, and other potential sources of bias. Each domain was rated as having low, unclear, or high risk of bias, and the overall risk was judged accordingly.

Results

**Figure 1:**
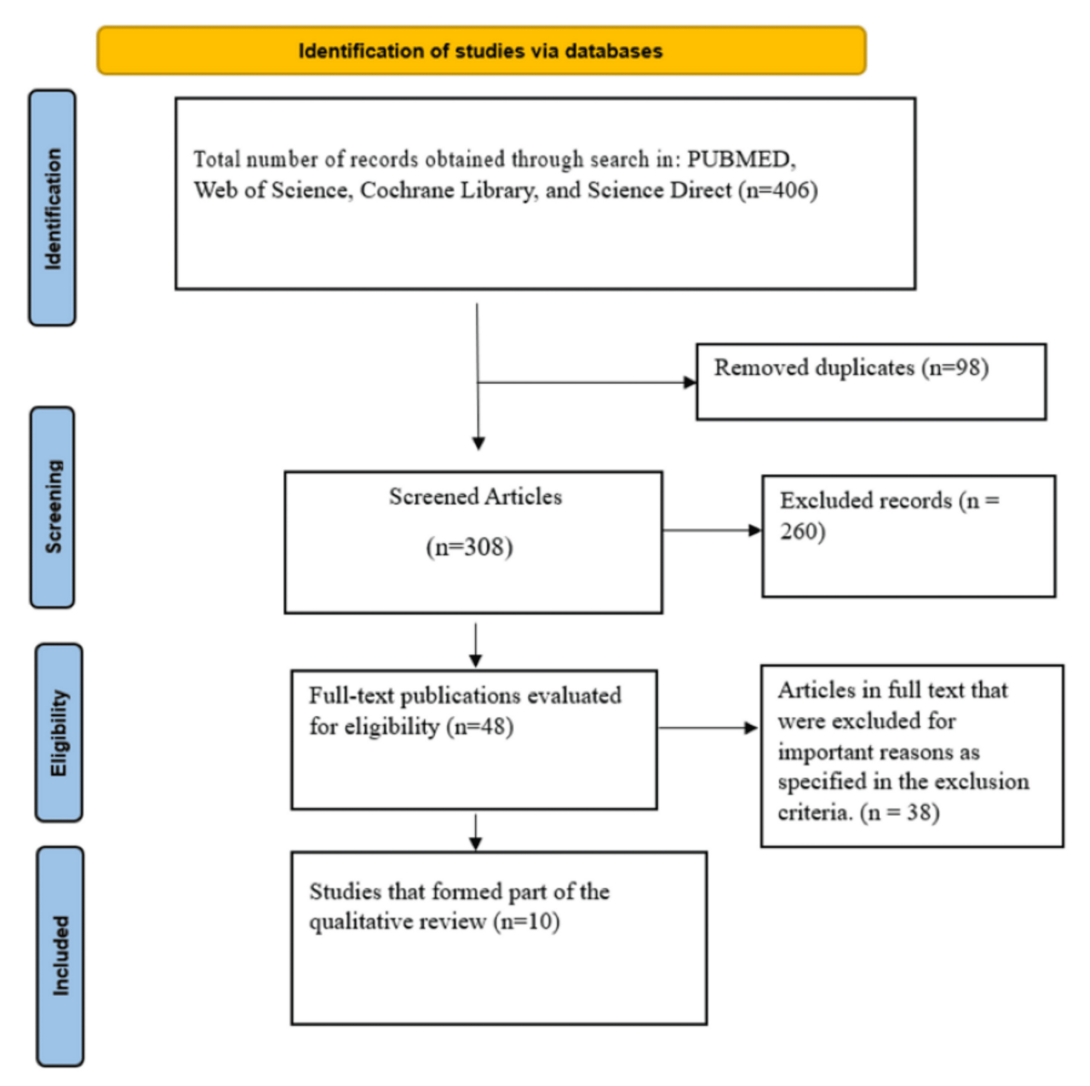
PRISMA Flow Diagram PRISMA, Preferred Reporting Items for Systematic Reviews and Meta-Analyses

There were 406 articles found in the initial search from the various databases: PUBMED (217), Web of Science (146), Cochrane Library (4), and ScienceDirect (39). The studies were then screened through the above eligibility criteria, with a final number of 10 studies eligible for inclusion and analysis, as illustrated in Figure 1.

Study Characteristics

The essential attributes of the studies included for review are detailed in Table 1. All 10 selected publications examined the impact of lifestyle interventions (diet and exercise) on the progression of DR. The studies were conducted in various regions worldwide and were published in English (Table 1).

**Table 1:** Study Characteristics

Authors	Study type	Intervention	N	Outcome/Results	Conclusion
Pardhan et al. (2023) [11]	Randomized controlled trial	A culturally and linguistically appropriate patient-centered, target-driven lifestyle intervention with video education training, dietary modifications, and physical activity guidance. Compliance was monitored weekly via telephone calls.	110	The intervention group experienced a significant reduction in HbA1c levels (7.2% to 6.1%), compared to a decrease from 7.1% to 6.6% in the control group (p < 0.05). Additionally, total cholesterol levels were lower in the intervention group (174 mg/dL vs. 186 mg/dL, p < 0.05), along with low-density lipoprotein (LDL) levels (95.5 mg/dL vs. 107.5 mg/dL, p < 0.05). White rice consumption decreased by 36.5% in the intervention group compared to only 4% in the control group (p < 0.05), while physical activity significantly increased in the intervention group (p < 0.05). Notably, 100% of participants in the intervention group attended diabetic retinopathy (DR) screening, whereas none in the control group did.	A culturally tailored, target-driven lifestyle intervention with video education significantly improved diabetes control, self-care, and health literacy in newly diagnosed diabetic patients. The approach could be scaled up for nationwide implementation to reduce diabetes-related complications and improve patient outcomes.
Mirghani et al. (2021) [12]	Cross-sectional study	Assessment of diet soda and non-nutritive sweetener consumption and their association with diabetic retinopathy and glycemic control.	200	A total of 33% of participants had diabetic retinopathy, while 70% had poorly controlled diabetes with HbA1c levels above 8%. Diet soda consumption was significantly associated with higher HbA1c levels and an increased prevalence of diabetic retinopathy (p < 0.05). However, non-nutritive sweeteners were linked to obesity but showed no association with retinopathy or HbA1c levels.	Diet soda consumption was linked to worse glycemic control and diabetic retinopathy, whereas non-nutritive sweeteners were associated with obesity. The study suggests further research is needed to explore the long-term impact of these dietary choices.
Pereira-Payo et al. (2024) [13]	Cross-sectional study using NHANES (National Health and Nutrition Examination Survey) data (2011-2020)	Analysis of lifestyle factors (sleep duration, physical activity, diet, sedentary behavior) and demographic variables affecting diabetic eye disease.	2,657	A higher risk of diabetic eye disease was observed in physically inactive individuals (OR: 1.48, RR: 1.36), males compared to females (OR: 1.77, RR: 1.61), those sleeping less than six hours or more than nine hours per night (OR: 1.61, RR: 1.43), and individuals over 62 years old (OR: 1.53, RR: 1.40). While diet and sedentary time were significant factors, they had a lesser influence compared to sleep and physical activity. Alcohol consumption was not found to be a significant factor for diabetic eye disease.	Sleep duration and physical activity are key modifiable factors influencing diabetic eye disease. Regular physical activity and maintaining 6-8 hours of sleep may lower the risk. Public health strategies should emphasize these lifestyle factors in diabetes management.
Zhao et al. (2020) [14]	Randomized controlled trial	Oral administration of *Abelmoschus manihot* (Jiahua tablets) alongside standard diabetic retinopathy care (blood glucose, blood pressure, and lipid management, exercise, and health education) for 6 months.	80	The severity of diabetic retinopathy improved in 25.4% of the treatment group compared to 9.3% in the control group (p = 0.01), while the progression rate was significantly lower at 4.2% in the treatment group versus 18.7% in the control group (p = 0.007). ETDRS (Early Treatment Diabetic Retinopathy Study) vision scores showed significant improvement in the treatment group (p < 0.001), and macular thickness, as measured by optical coherence tomography (OCT), demonstrated a significant reduction in cube average thickness, central subfield thickness, and cube volume in the treatment group (p < 0.0001). Additionally, serum vascular endothelial growth factor (VEGF) levels were significantly lower in the treatment group after six months (p = 0.0026).	*Abelmoschus manihot* (Jiahua tablets) showed significant improvement in non-proliferative diabetic retinopathy (NPDR) severity, vision scores, macular thickness, and VEGF reduction. It may serve as a novel complementary treatment for diabetic retinopathy.
Yao et al. (2024) [15]	Randomized controlled trial	Internet + E-Coach chronic disease management system, including daily medication reminders, personalized diet and exercise guidance, health education, and remote consultations.	208	After 12 months, fasting plasma glucose (FPG), 2-hour postprandial glucose (2hPG), triglycerides (TG), and alanine transaminase (ALT) levels were significantly lower in the intervention group (p < 0.05). Inflammatory markers, including IL-6, TNF-α, and high-sensitivity C-reactive protein (Hs-CRP), were also significantly reduced (p < 0.05). Quality of life, as measured by CLVQOL (Chinese Low Vision Quality of Life) scores, showed significant improvements in visual function, mobility, and daily activities (p < 0.05). Additionally, participants in the intervention group demonstrated improved disease knowledge and greater adherence to healthy behaviors.	The Internet + E-Coach system improved glycemic control, reduced inflammation, and enhanced quality of life in chronic DR patients compared to conventional care. Digital interventions may be an effective approach to chronic disease management.
Loprinzi (2015) [16]	Cross-sectional study (NHANES 2005-2006)	Examined the effects of physical activity and healthy eating on diabetic retinopathy. Physical activity was measured via accelerometry, and diet was assessed using the Healthy Eating Index.	223	Physical activity (OR = 0.70, p = 0.42) and healthy eating (OR = 0.36, p = 0.16) were not independently associated with moderate-to-severe diabetic retinopathy. However, individuals who engaged in both behaviors had significantly reduced odds of developing moderate-to-severe diabetic retinopathy (OR = 0.03, p = 0.02). The additive interaction effect indicated that the combined impact of physical activity and healthy eating was greater than the sum of their individual effects (AP = 0.57, p < 0.05).	Concurrent adoption of healthy eating and regular physical activity significantly reduces the risk of moderate-to-severe diabetic retinopathy, reinforcing the importance of holistic lifestyle interventions in diabetes management.
Aro et al. (2019) [17]	Prospective cohort study (Finnish Diabetes Prevention Study - DPS)	Lifestyle intervention including weight loss, healthy diet, and physical activity in individuals with impaired glucose tolerance (IGT).	211	Microaneurysm occurrence was lower in the intervention group (24%) compared to the control group (38%) (p = 0.029), with the intervention group showing a significantly reduced risk of microaneurysms (OR = 0.52, p = 0.039). Baseline triglyceride levels were significantly associated with microaneurysm development (p = 0.003), while no significant differences were observed in other retinal changes between the groups.	Intensive lifestyle intervention in individuals with impaired glucose tolerance reduced the occurrence of early diabetic microvascular changes (microaneurysms). Serum triglycerides were a key predictor of early retinal changes, suggesting their role in diabetic retinopathy development.
Kaarniranta et al. (2025) [18]	Long-term cohort study (Finnish Diabetes Prevention Study - DPS)	Intensive lifestyle intervention (weight loss, healthy diet, and physical activity) in individuals with impaired glucose tolerance (IGT) vs. usual care.	505	There was no significant difference in the cumulative incidence of clinically diagnosed diabetic retinopathy (DR) between the intervention group (8.9%) and the control group (7.7%) (OR = 1.15, 95% CI 0.61-2.21). However, a higher cumulative HbA1c was significantly associated with an increased risk of DR (HR = 1.4, 95% CI 1.02-1.88). Additionally, a diabetes diagnosis during follow-up was linked to a higher risk of DR (OR = 1.81, 95% CI 0.86-4.19), though this association was not statistically significant.	Long-term lifestyle intervention did not reduce the incidence of clinically diagnosed DR. Instead, higher cumulative HbA1c was the strongest predictor of DR risk. Effective glycemic control remains the key preventive strategy against DR.
Gong et al. (2010) [19]	Long-term prospective cohort study (China Da Qing Diabetes Prevention Outcome Study)	A 6-year lifestyle intervention (diet, exercise, and diet + exercise) in individuals with impaired glucose tolerance (IGT) followed for 20 years	577	The cumulative incidence of severe diabetic retinopathy (DR) was lower in the intervention group (9.2%) compared to the control group (16.2%) (p = 0.03), with the intervention group showing a 47% reduced risk of severe DR (HR = 0.53, 95% CI 0.29-0.99, p = 0.048). However, there were no significant differences between the groups in the incidence of nephropathy (HR = 1.05, p = 0.96) or neuropathy (p = 0.89). Higher cumulative HbA1c levels and longer diabetes duration were associated with an increased risk of DR.	A 6-year lifestyle intervention in IGT patients reduced the incidence of severe, vision-threatening DR by 47% over 20 years, likely due to delayed diabetes onset. However, similar benefits were not observed for nephropathy or neuropathy. This highlights the long-term importance of lifestyle interventions in reducing DR risk.
Qin et al. (2024) [20]	Longitudinal cohort study (cross-sectional and prospective)	Gut microbiota profiling, dietary investigations, and metabolite analysis in diabetic patients with and without diabetic retinopathy (DR)	99	Diabetic retinopathy (DR) patients exhibited distinct gut microbiome signatures, characterized by lower levels of Butyricicoccus and the Ruminococcus torques group, along with alterations in metabolic pathways. Lower plasma acetate and butyrate levels were correlated with a higher risk of DR. Machine learning models utilizing gut microbiome data accurately predicted DR development (AUC = 0.951). Additionally, high dietary cholesterol intake and reduced fat energy expenditure were associated with an increased risk of DR.	Gut microbiota composition, particularly short-chain fatty acid (SCFA)-producing bacteria, plays a role in diabetic retinopathy (DR) risk. Gut microbiome-based biomarkers could predict DR onset, providing potential targets for early intervention.

Risk of Bias Assessment

To assess the quality of the cross-sectional, cohort, and retrospective/prospective studies, the NOS was used (Table 2). Of the seven studies evaluated, five were found to have a low risk of bias and were deemed to be of excellent quality [12,13,17,19,20]. However, two out of seven studies had a moderate risk of bias [16,18]. Overall, the included studies were of good quality (Table 2).

**Table 2:** Newcastle Ottawa Quality Assessment Scale (NOS) A study was given up to one star (*) for each numbered item in the outcome and selection categories. Comparability, however, was rated with up to two stars (**), according to the evaluation's findings. * indicates a low risk of bias in both selection and outcome. In terms of comparability, ** indicates a low risk of bias, whereas (-) indicates a significant risk of bias.

Author	Selection				Comparability	Outcome			Total score (1-3 high risk; 4-6 moderate risk; 7-9 low risk)
	Representativeness of the exposed cohort	Selection of the non-exposed cohort	Ascertainment of exposure	Demonstration that the outcome of interest was not present at the start of the study	Comparability of cohorts on the basis of the design or analysis	Assessment of outcome	Was the follow-up long enough for outcomes to occur	Adequacy of follow-up of cohorts	
Mirghani et al. (2021) [12]	*	-	*	*	**	*	*	*	8/9
Pereira-Payo et al. (2024) [13]	*	-	*	*	**	*	*	-	7/8
Loprinzi (2015) [16]	*	-	*	*	*	*	*	-	6/9
Aro et al. (2019) [17]	*	-	*	*	**	*	*	-	7/8
Kaarniranta et al. (2025) [18]	*	-	*	*	*	*	*	-	6/9
Gong et al. (2010) [19]	*	-	*	*	**	*	*	*	8/9
Qin et al. (2024) [20]	*	-	*	*	**	*	*	-	7/9

**Figure 2:**
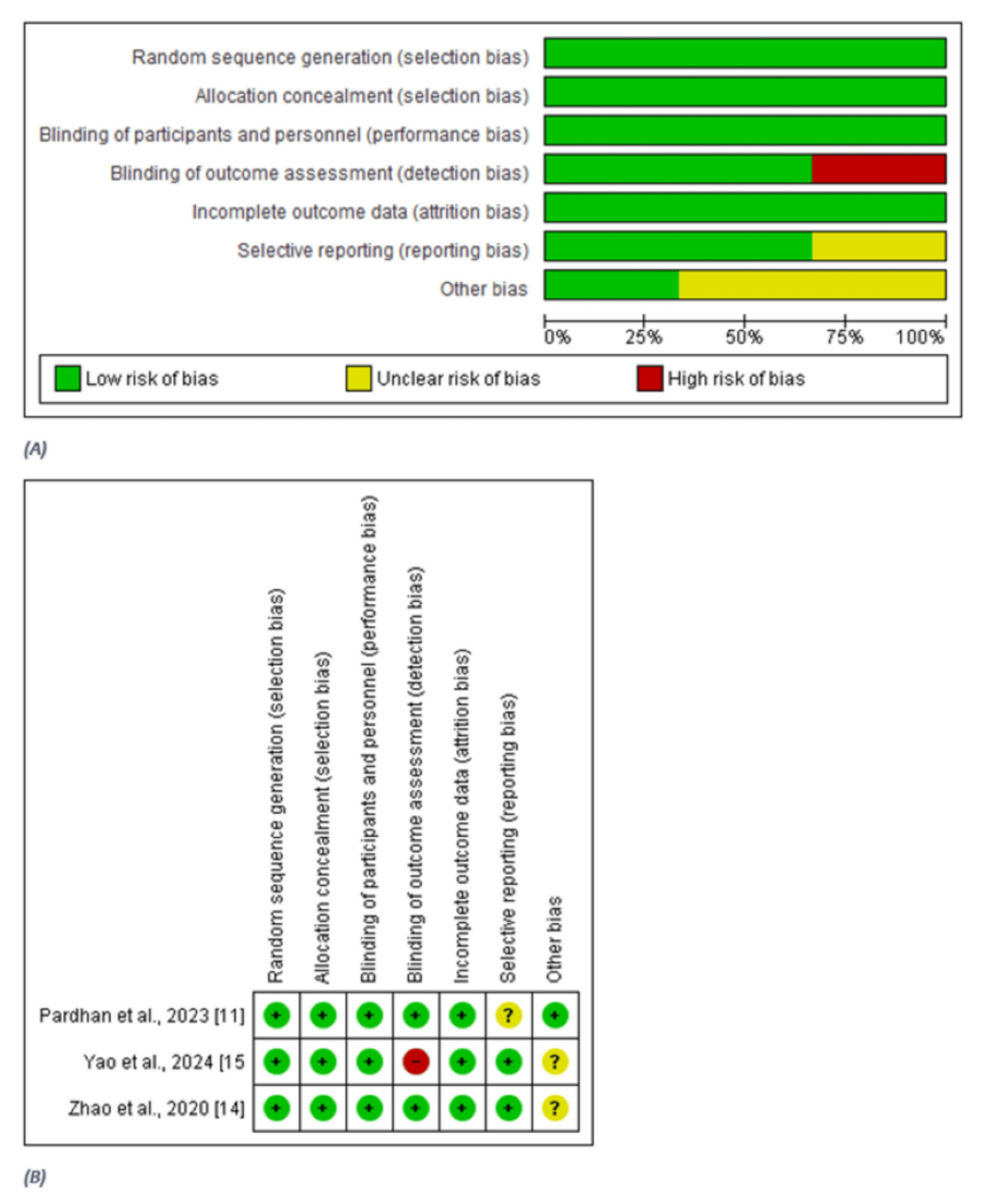
Cochrane Risk of Bias Assessment Figure A shows the overall risk of bias summary across the included randomized controlled trials (RCTs). Most domains showed a low risk of bias; however, there was a high risk in the domain of blinding of outcome assessment, and unclear risks in selective reporting and other bias domains. Figure B details study-specific assessments. Yao et al. (2024) showed high risk for outcome assessment blinding and unclear other bias [15]. Pardhan et al. (2023) had unclear selective reporting bias [11], while Zhao et al. (2020) showed unclear risk in the other bias domain [14].

The risk of bias for the included RCTs was assessed using the Cochrane Risk of Bias Assessment Scale. The results reveal that 4/7 items in the included studies had a low risk of bias [11,14,15], 2/7 items had an unclear risk of bias [11,14,15], and 1/7 items had a high risk of bias [15]. However, overall, all studies were of good quality (Figure 2).

Discussion

Diabetes mellitus is a growing global health concern that requires the adoption of appropriate preventive measures, including, but not limited to, managing blood pressure, lipid profiles, and glycemia; promoting a healthy lifestyle; modifying dietary habits; and increasing physical activity [2]. Based on this premise, this systematic review focused on assessing the impact of lifestyle interventions (diet and exercise) on the progression of DR in patients diagnosed with diabetes mellitus. 

Dietary interventions, especially those emphasizing low-glycemic index (GI) foods, plant-based diets, or Mediterranean-style diets, showed a positive influence on both glycemic control and inflammatory pathways. These dietary patterns were linked to stabilization of DR severity, improved metabolic outcomes, and, in some cases, delayed progression to proliferative stages of DR [13,15]. Additionally, consumption of white rice, increased physical activity, improved lipid profiles, reduced total and low-density lipoprotein (LDL) cholesterol, and lower HbA1c values were found to positively impact the reduction of DR progression. Health awareness and disease management were also improved by the intervention, as evidenced by higher adherence to DR screening in the intervention group [11]. According to Mirghani et al., consuming sugary beverages, such as soda, was associated with higher HbA1c levels and an increased incidence of DR, suggesting that it may have a detrimental effect on diabetes management and its complications [12]. 

A study by Pereira-Payo et al. reported that individuals who are physically inactive - particularly men - and those who get less than six hours of sleep per night are more likely to develop DR. The study noted that sleep and physical activity had a greater impact than diet and sedentary behavior, while alcohol consumption was not identified as a significant risk factor. It emphasized that reducing the risk of DR requires regular exercise and adequate sleep [13]. Zhao et al. reported that *Abelmoschus manihot*, also known as Jiahua tablets, may be a viable adjunctive treatment for non-proliferative diabetic retinopathy (NPDR), as it enhances retinal structure and vision, and decreases disease severity. The intervention significantly improved patients' visual function, retinal structural alterations, disease progression, and DR severity. Additionally, the treatment resulted in decreased serum VEGF levels, which may indicate a mechanism for reduced retinal vascular injury. However, the report suggested that further research is necessary to determine its role in the clinical treatment of diabetes [14].

In the study by Yao et al., quality of life (QoL) was assessed using the Chinese-version Low Vision Quality of Life Questionnaire (CLVQOL). Significant improvements were reported across multiple domains, including visual function, mobility, social interaction, and emotional well-being (p < 0.05), indicating that the digital intervention not only improved clinical markers but also enhanced patients' daily functioning and psychological health. Additionally, the system improved adherence to healthy habits and disease knowledge, demonstrating its potential as a scalable and effective strategy for managing chronic illnesses [15]. Loprinzi found that a holistic approach to diabetes management, including both physical activity and healthy eating, significantly reduced the risk of developing moderate-to-severe DR. The combined impact of these factors was greater than that of individual factors, suggesting a synergistic benefit. Therefore, promoting healthy eating and regular physical activity is crucial for diabetes management [16]. 

Physical activity interventions varied in duration, intensity, and modality. Studies implementing moderate-intensity aerobic exercise for at least 150 minutes per week, consistent with WHO and ADA guidelines, reported improvements in glycemic control (HbA1c reduction), reductions in systemic inflammation (e.g., CRP and IL-6), and decreased oxidative stress biomarkers - all of which are known contributors to DR progression [15,18,20]. For instance, structured walking programs or combined aerobic-resistance training were associated with slower DR progression, improved retinal microvasculature integrity, and better visual acuity outcomes in specific cohorts [15,20].

A study by Aro et al. indicated that individuals with impaired glucose tolerance experienced a 52% decrease in microaneurysm incidence after undergoing an intensive lifestyle intervention. The study also highlighted the importance of lipoprotein control in slowing the progression of DR, as baseline triglyceride levels were demonstrated to be a major predictor of microaneurysm development. It emphasized the importance of implementing early preventive strategies for individuals at risk of diabetes [17]. In contrast, a study by Kaarniranta et al. reported no significant long-term impact of lifestyle changes on the incidence of clinically diagnosed DR. However, the study found that elevated cumulative HbA1c levels were a significant indicator of an increased risk for DR. It also noted that individuals diagnosed with diabetes during follow-up had a higher risk of developing DR. Therefore, the study recommended that maintaining blood sugar control is crucial in regulating the rate of DR progression [18]. 

A study by Gong et al. reported that individuals with impaired glucose tolerance who participated in a six-year lifestyle intervention had a considerably lower incidence of severe DR over a 20-year period. The reduced risk of developing severe DR in the intervention group suggests that lifestyle changes can slow disease progression [19]. Additionally, Qin et al. found that the risk of DR is greatly influenced by the composition of gut microbiota, particularly bacteria that produce short-chain fatty acids. The levels of these bacteria, as well as plasma acetate and butyrate, were lower in DR patients. The potential for microbiome-based biomarkers in early diagnosis was demonstrated through a machine learning algorithm that accurately predicted the onset of DR [20]. Although the gut microbiota-based prediction model demonstrated high accuracy in identifying DR risk (AUC = 0.951), these findings should be interpreted as emerging evidence. Given the relatively small sample size and early-stage nature of this research, these results remain hypothesis-generating and warrant further validation through larger, longitudinal cohort studies.

The evidence strongly supports the integration of structured lifestyle interventions into clinical practice for DR management. Healthcare providers should consider prescribing individualized physical activity routines, ideally reaching or exceeding 150 minutes of moderate aerobic exercise per week [15,20]. Notably, combined interventions - incorporating both diet and exercise - demonstrated more substantial benefits compared to single-modality approaches, underlining the need for a comprehensive lifestyle strategy [15,20].

Despite the relatively low heterogeneity of the findings in the studies included in this systematic review, several limitations were noted. These included short follow-up periods in some studies, variations in study design, reliance on self-reported data, small sample sizes in some studies, and the presence of confounding variables - all of which increased the risk of recall bias. Despite these limitations, the findings of this study provide valuable insights and are sufficient to draw meaningful conclusions. However, further research is needed to strengthen the evidence base and enhance clinical recommendations. 

## Conclusions

This comprehensive review highlights the importance of lifestyle changes, dietary regulations, metabolic factors, and gut microbiota control in preventing and managing DR. It suggests that technology-driven, culturally relevant interventions can improve general health literacy, diabetes management, and self-care practices, leading to better metabolic outcomes and a reduced risk of DR. The findings demonstrate that lifestyle modifications contribute to improved glycemic control, reduced inflammation, lower retinal vascular complications, and enhanced overall diabetes management. In terms of lowering the risk of moderate-to-severe DR, lifestyle changes such as regular exercise and a balanced diet are more beneficial when combined, rather than practiced individually. On that basis, future RCTs should explore the implementation of culturally tailored, technology-assisted lifestyle intervention platforms, particularly in low-resource and underserved settings, where the burden of DR is increasing but access to structured prevention programs remains limited.
